# Identification of primary retinal cells and ex vivo detection of proinflammatory molecules using flow cytometry

**Published:** 2009-07-17

**Authors:** Jose-Andres C. Portillo, Genevieve Okenka, Timothy S. Kern, Carlos S. Subauste

**Affiliations:** 1 Department of Ophthalmology and Visual Sciences, Case Western Reserve University School of Medicine, Cleveland, OH; 2 Department of Medicine, Case Western Reserve University School of Medicine, Cleveland, OH; 3 Veterans Administration Medical Center, Research Service (151), Cleveland, OH; 4 Department of Pathology, Case Western Reserve University School of Medicine, Cleveland, OH

## Abstract

**Purpose:**

Advances in the understanding of the pathogenesis of retinal disorders can be facilitated by a methodology to measure expression of proinflammatory molecules in various subsets of retinal cells.

**Methods:**

We examined whether a multiparameter flow cytometric assay can be used to identify various subsets of retinal cells and examine expression of molecules involved in inflammatory responses in the retina. Single-cell suspensions freshly obtained after enzymatic digestion of normal mouse retinas were stained with antibodies against cluster of differentiation 11b (CD11b), cluster of differentiation 31 (CD31), Glial fibrillary acidic protein (GFAP), rhodopsin, Thy-1, and vimentin. These markers were previously shown by immunohistochemistry to label retinal microglia/macrophages, endothelial cells, astrocytes, photoreceptors, ganglion neurons, and Müller cells respectively in normal mouse retinas.

**Results:**

Costaining with antibodies against intercellular adhesion molecule-1 (ICAM-1) and CD40 revealed that ICAM-1 is normally expressed at various levels on all subsets of retinal cells examined. In contrast, CD40 was detected only on CD11b^+^, CD31^+^, Thy-1^+^, and vimentin^+^ cells. Ischemia-reperfusion of the retina resulted in upregulation of ICAM-1 on CD105^+^ and vimentin^+^ cells and upregulation of nitric oxide synthase 2 in CD11b^+^ cells.

**Discussion:**

These results indicate that flow cytometry can be used to readily quantitate expression of surface and intracellular molecules of relevance to retinopathies in freshly isolated retinal cells.

## Introduction

Increasing evidence indicates that inflammation is an important component of the pathogenesis of retinopathies [1,2]. Intercellular adhesion molecule-1 (ICAM-1 or CD54) and nitric oxide synthase 2 (NOS2) are two molecules involved in retinal disorders. ICAM-1 is an adhesion molecule expressed on endothelial cells and leukocytes that participates in the recruitment of leukocytes to sites of inflammation. Inflammatory infiltration in the inner retina leads to retinal injury after ischemia and reperfusion (I-R) [3]. ICAM-1 is upregulated in the ischemic retina [1] and administration of anti-ICAM-1 monoclonal antibody (mAb) partially prevents injury to the inner retina [3]. In the case of diabetic retinopathy, the retinal vasculature expresses higher levels of ICAM-1 [4]. Moreover, blockade of ICAM-1 prevents diabetic retinal leukostasis, vascular leakage, and vascular histopathology [2,5].

Inflammatory stimuli in acute retinal ischemia induce NOS2 [1]. Similarly, NOS2 is upregulated in diabetic retinopathy [6]. Induction of NOS2 is relevant because administration of an NOS2 inhibitor partially protects against retinal thinning after ischemia and diabetic retinopathy [7,8], and NOS2^−/−^ mice are resistant to diabetic retinopathy [9].

CD40 is an important regulator of inflammation. CD40 is a member of the TNF receptor superfamily that is expressed both on antigen-presenting cells and various nonhematopoietic cells [10-13]. Its counter-receptor, CD154 (CD40 ligand), is expressed on activated CD4^+^ T cells and platelets, and is also present in soluble form in plasma [10,11]. CD40-CD154 interaction is central to regulation of both cellular and humoral immunity [10,11,14]. In addition, CD40-CD154 signaling activates inflammation. Indeed, the CD40-CD154 pathway is pivotal for the development and progression of atherosclerosis, graft rejection, and various autoimmune disorders [15]. Recent studies uncovered that retinal inflammatory infiltration and neurovascular degeneration after acute retinal ischemia are driven by CD40 [16].

Despite important advances, we still have an incomplete understanding of the pathogenesis of retinopathies. An assay that can readily quantitate expression of surface and intracellular molecules involved in retinal injury would further our understanding of the pathogenesis of various forms of retinal diseases. Herein we report on a flow cytometric method to identify various retinal cell subsets and quantitate proinflammatory molecules in these cells.

## Methods

### Animals

Male C57BL/6 (B6) mice were obtained from Jackson Laboratories (Bar Harbor, ME). Animals had a weight of 25 to 30 g (10–14 week) when used for experiments. Experiments were approved by the Institutional Animal Care and Use Committee of Case Western Reserve University School of Medicine.

### Isolation of primary retinal cells

Mice were anesthetized and perfused through the heart with phosphate buffer serum (PBS, Mediatech, Manassas, VA) to remove blood from the eyes before organ collection. Mice were anesthetized by intramuscular injection of 0.15 ml of Triple Cocktail containing ketamine, xylazine, and acepromazine. This was followed by perfusion through the heart. Retinas were isolated and minced following by digestion in a solution containing 15 IU/ml papain and 15 μg/ml DNase (Worthington Biochemicals, Freehold, NJ) for 30 min at 37 °C. Tissue was dissociated by gentle pipetting and passed through a 40 μm cell strainer. Flow-through was mixed with Fetal bovine serum (FBS; HyClone Laboratories Inc. South Logan, UT) and washed. Tissue trapped by the strainer was digested with 1 mg/ml collagenase type I (Worthington Biochemicals) for 30 min at 37 °C to free endothelial cells. After dissociation and mixing with FBS, cells were washed once in DMEM (Mediatech) with 10% FBS for 5 min at 300 g at room temperature. Cells obtained after papain-DNase and collagenase treatments were pooled and counted. Viability of the cells was consistently greater than 90% as assessed by trypan blue exclusion. Cells were kept on ice in Ca^2+^, Mg^2+^-free PBS (Mediatech) until immunofluorescent labeling.

### Flow cytometry

Suspensions of primary retinal cells from two to five mice were pooled and were incubated for 10 min with immunoglobulin constant Fragment (Fc)-receptor blocking antibody (Fc block: anti-CD16/CD32, clone 2.4G2; BD Biosciences, San Jose, CA) to reduce nonspecific binding of antibodies used for flow cytometry. Retinal cell suspensions were incubated with the following antibodies: allophycocyanin (APC)-conjugated or APC-Alexa Fluor 750-conjugated anti-CD11b (this marker identifies microglia in the normal retina; eBiosciences, San Diego, CA), APC-conjugated anti-CD31 (to detect endothelial cells; eBiosciences), APC-conjugated anti-CD105 (to detect endothelial cells; R&D Systems, Minneapolis, MN), APC-conjugated anti-Thy1 (to detect ganglion neurons; eBiosciences) or appropriate isotype control antibodies. For detection of intracellular markers, cells were fixed and permeabilized by using IntraPrep permeabilization reagent (Beckman Coulter, Hialeah, FL) following manufacturer’s instructions [17]. Thereafter, cells were stained with fluorescein isothiocyanate (FITC)-conjugated anti-vimentin (to detect Müller cells; Santa Cruz Biotechnologies, Santa Cruz, CA), phycoerythrin (PE)-conjugated anti-glial fibrillary acidic protein (GFAP; this marker identifies astrocytes in the normal mouse retina; Santa Cruz), anti-rhodopsin mAb (to detect photoreceptors; 1D4 and C7, gifts from Dr. Krzysztof Palczewski, Case Western Reserve University, Cleveland, OH) followed by APC-conjugated donkey F(ab’)2 fragment anti-mouse IgG Ab (eBiosciences) or appropriate isotype control Abs. Cells were analyzed after fixation with 1% paraformaldehyde. In certain experiments, cells were jointly stained with PE-conjugated or FITC-conjugated anti-CD40 mAb (BD Biosciences), PE-conjugated or FITC-conjugated anti-ICAM-1 mAb (eBiosciences) or Alexa Fluor 647-conjugated anti-NOS2 Ab (Santa Cruz). Expression of CD40 or ICAM-1 was analyzed on gated CD11b^+^, CD31^+^, CD105^+^, GFAP^+^, rhodopsin^+^, vimentin^+^, or Thy-1^+^ cells. Expression of NOS2 was analyzed in permeabilized CD11b^+^ cells. The average levels of expression of CD40, ICAM-1, and NOS2 in various retinal cell subsets are expressed as corrected Mean Fluorescence Intensity (cMFI). This was obtained by subtracting fluorescence obtained with isotype control Ab from the fluorescence obtained with Abs against CD40, ICAM-1, or NOS2. Flow cytometry data acquisition was performed using a LSR II and running FACSDiva software (Becton Dickinson, San Jose, CA). FlowJo software (Tree Star Inc., Ashland, OR) was used for data analysis. In certain experiments, vimentin^+^, and rhodopsin high (rhodopsin^hi^) events were sorted using a FACSAria (Becton Dickinson). Sorted events were analyzed by light microscopy.

Mouse endothelial cells were used to determine whether enzymatic treatment affects expression of surface markers. Mouse high endothelial venule cells c (mHEVc) [18] stably transfected with CD40-encoding plasmid [19] were detached by exposure to Versene (Invitrogen, Carlsbad, CA) for 5 min. Cell suspensions were then incubated with or without enzymatic cocktail as we have described in this paper. Cells were subsequently incubated with fluorescent antibodies and were subjected to flow cytometry analysis

### Model of retinal ischemia and reperfusion

Retinal ischemia was induced as we previously described [1,16]. The anterior chamber of one eye was cannulated with a 30 gauge needle attached to a line infusing normal saline. Intraocular pressure (IOP) was measured by a handheld tonometer (TONO Pen; Medtronic Solan, Jacksonville, FL) and pressure in the eye was regulated to 80 mmHg to 90 mmHg with a pressure infuser (Infu-surg; Ethox Corp., Buffalo, NY) and maintained for 90 min. The other eye of the same animal was set up as a control. After ischemia, the needle was withdrawn, IOP was normalized, and reflow of the retinal circulation was documented visually. Mice were euthanized with triple cocktail one or seven days after I-R injury and retinal cell suspensions were processed for flow cytometry.

### Statistical analysis

All results were expressed as the mean±standard error of the mean. Data were analyzed by two-tailed Student’s *t* test. Differences were considered statistically significant at p<0.05.

## Results

### Detection of ICAM-1 and CD40 on various retinal cells by flow cytometry

Enzymatic digestion was performed to obtain retinal single cell suspensions. Pilot experiments were conducted to determine whether enzymatic treatment impairs expression of surface markers. Cell suspensions of CD40^+^ mouse endothelial cells (mHEVc) were treated with or without enzymatic cocktail as described in Methods followed by flow cytometric analysis of cell surface markers. The levels of expression (cMFI) of CD31, CD40 and ICAM-1 were not affected by enzymatic treatment. The levels of expression for these molecules were as follows: No enzymatic treatment: CD31=105±14, CD40=99±7, ICAM-1=82±4; Enzymatic treatment: CD31=100±1, CD40=82±8, ICAM-1=78±1 (n=3; p>0.5). Thus, the protocol of enzymatic digestion appeared unlikely to significantly affect expression of surface molecules.

**Figure 1:**
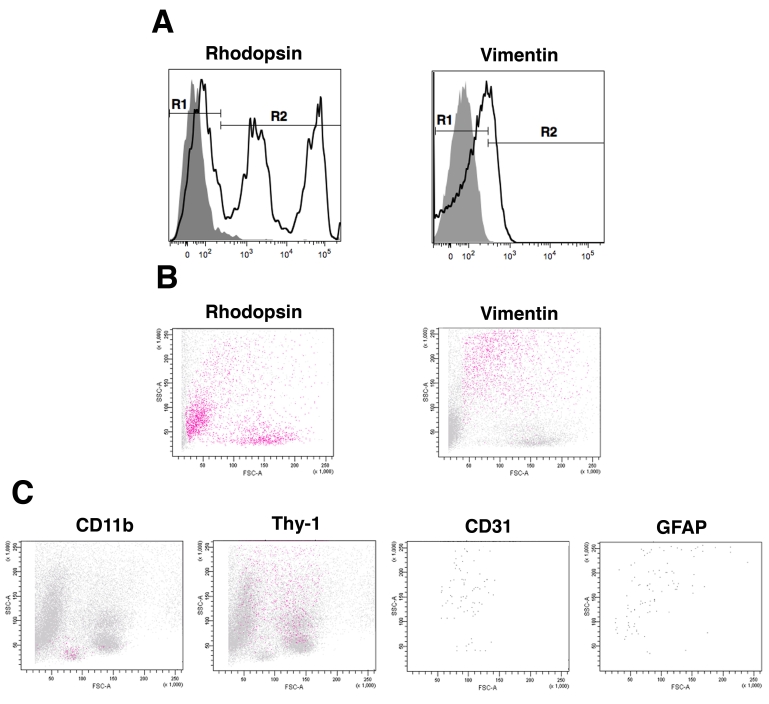
Flow cytometric analysis of retinal cell suspensions. **A:** Single-cell suspensions obtained after enzymatic digestion of retinas were permeabilized and stained with antibodies against rhodopsin and vimentin (thick lines) or isotype control antibodies (shaded areas) as described in Methods. The gate R2 in histograms reveal events that stained with either anti-rhodopsin or anti-vimentin antibodies. **B:** The forward scatter (FSC) versus side scatter (SSC) profile of retinal cells is shown. Red dots represent vimentin^+^ or rhodopsin^+^ events as defined by gating criteria (R2) shown in panel **A. C:** Single-cell suspensions were stained with antibodies against CD11b, CD31, and Thy-1 to detect surface expression of these molecules. Permeabilized cells were incubated with anti-GFAP antibody. Red dots represent events that stained with either CD11b or Thy-1 as indicated. Due to low frequency of CD31^+^ and GFAP^+^ events, cells that did not stain with anti-CD31 and anti-GFAP antibodies were removed from the dot plots, thus enabling easier identification of CD31^+^ and GFAP^+^ events. Results are representative of six to nine independent experiments.

**Figure 2:**
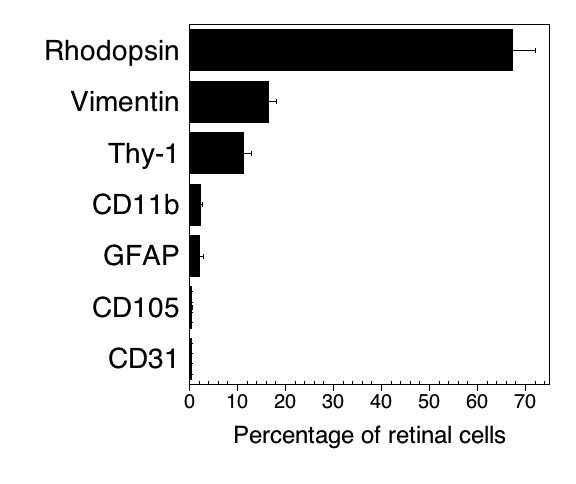
Phenotypic composition of retinal cell suspensions. Single-cell suspensions were stained with the indicated antibodies. Data are shown as the mean±SEM from six to nine independent experiments.

Single-cell suspensions were obtained from exsanguinated retinas. Each retina yielded on average 2.01±0.20x10^6^ cells (n=15). We applied flow cytometry to identify various cell types in the normal mouse retina. We used a panel of antibodies against markers shown by immunofluorescence to be expressed by different cell subsets in the normal mouse retina: CD11b for microglia/macrophages [20], CD31 for endothelial cells [21], GFAP for astrocytes [21], rhodopsin for photoreceptors [22,23], Thy-1 for ganglion neurons [24], and vimentin for Müller cells [21]. We started by examining photoreceptors and Müller cells, two major cell types in the retina. Retinal cells were permeabilized to detect intracellular rhodopsin and vimentin. Staining with anti-rhodopsin or anti-vimentin antibodies revealed expression of these intracellular markers as shown in Figure 1A. Regardless of the anti-rhodopsin antibody used (1D4 or C7), the expression of rhodopsin was bimodal (Figure 1A). Sorting of rhodopsin^hi^ events revealed that they consisted of rod-like structures of 1 μm in diameter (not shown), morphological features consistent with outer segments, the portion of the photoreceptors that expresses high levels of rhodopsin. Flow cytometry also examines two forms of light scatter: forward scatter (FSC) and side scatter (SSC), parameters that indicate the size and cell granularity respectively. Figure 1B reveals the FSC and SSC pattern of rhodopsin^+^ and vimentin^+^ retinal cells. Similarly, gating on CD11b^+^, CD31^+^, GFAP^+^, and Thy-1^+^ cells revealed the various patterns of FSC and SSC for each cell population (Figure 1C). The phenotypic composition of the cells released from the retina after enzymatic digestion is shown in Figure 2. Results were similar regardless of whether surface markers were assessed on permeabilized versus non-permeabilized cells (not shown). These studies also include staining with a mAb against CD105, a marker for endothelial cells.

**Figure 3:**
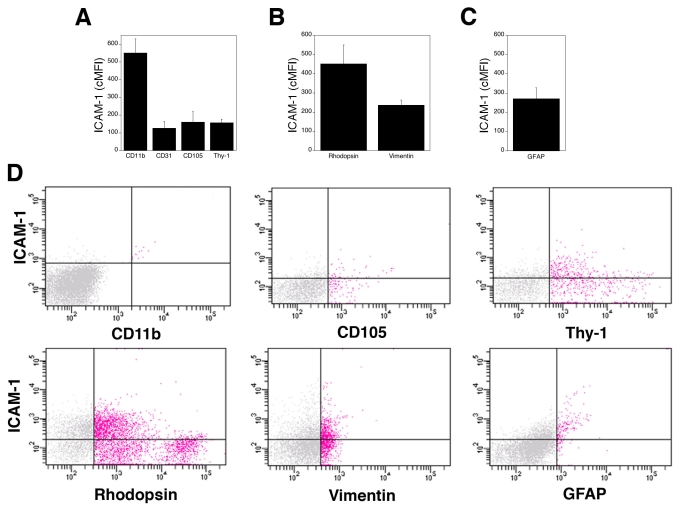
Expression of ICAM-1 on various subsets of retinal cells. Single-cell suspensions were stained with isotype control or with anti-ICAM-1 mAb. Cells were coincubated with antibodies that detect cell surface expression of CD11b, CD31, CD105, or Thy-1 (**A**), or cells were permeabilized followed by incubation with antibodies against rhodopsin and vimentin (**B**) or GFAP (**C**) to detect intracellular expression of these markers. PE-conjugated anti-ICAM-1 mAb was used in all conditions except when staining GFAP^+^ cells, where FITC-conjugated mAb was used instead. The average expression of ICAM-1 (cMFI) on gated CD11b^+^, CD31^+^, CD105^+^, Thy-1^+^, GFAP^+^, rhodopsin^+^, and vimentin^+^ events were calculated as described in Methods. Data are shown as the mean±SEM from three to four independent experiments. **D:** Representative dot plots of ICAM-1 versus various markers are shown. Red dots represent cells that expressed these markers. Horizontal and vertical lines represent gates obtained with isotype control antibodies.

**Figure 4:**
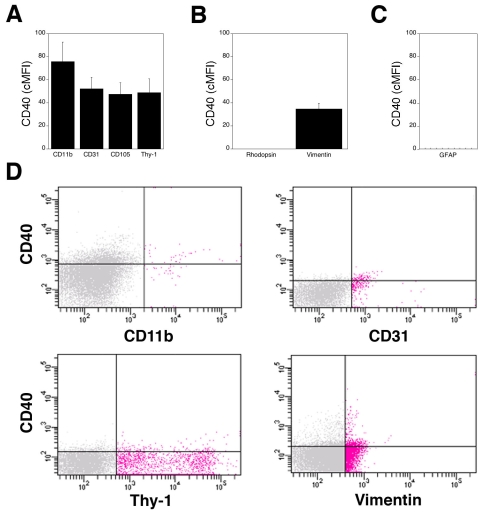
Expression of CD40 on various subsets of retinal cells. Single-cell suspensions were stained with isotype control or with anti-CD40 mAb. Cells were coincubated with antibodies that detect cell surface expression of CD11b, CD31, CD105, or Thy-1 (**A**), or cells were permeabilized followed by incubation with antibodies against rhodopsin and vimentin (**B**), or GFAP (**C**) to detect intracellular expression of these markers. PE-conjugated anti-CD40 mAb was used in all conditions except when staining GFAP^+^ cells, where FITC-conjugated mAb was used instead. The average expression of CD40 (cMFI) on gated CD11b^+^, CD31^+^, CD105^+^, Thy-1^+^, GFAP^+^, rhodopsin^+^, and vimentin^+^ events were calculated as described in Methods. Data are shown as the mean±SEM from three to four independent experiments. CD40 was not detected on rhodopsin^+^ and GFAP^+^ cells. **D:** Representative dot plots of CD40 versus various markers are shown. Red dots represent cells that expressed these markers. Horizontal and vertical lines represent gates obtained with isotype control antibodies.

ICAM-1 is an adhesion molecule that plays a pivotal role in recruitment of leukocytes to the retina and the development of vascular histopathology [2,5]. In addition, recent studies identified CD40 as an important regulator of retinal inflammation and neurovascular degeneration [16]. We used flow cytometry to examine the expression of these molecules on various retinal cells. Cell suspensions were stained with anti-ICAM-1 or anti-CD40 mAb plus either antibodies against CD11b, CD31, CD105, GFAP, rhodopsin, Thy-1, or vimentin. Varying levels of ICAM-1 were detected on all the subsets of retinal cells examined (Figure 3). ICAM-1 expression did not appear to be homogenous within rhodopsin^+^ and Thy-1^+^ cell populations. CD40 was detected only on CD11b^+^ (microglia/macrophages), CD31^+^(endothelial), Thy-1^+^(ganglion), and vimentin^+^ cells (Müller) cells (Figure 4). Despite the typical low levels of expression, CD40 is an important mediator of inflammation [15]. These results indicate that flow cytometry allows quantitation of surface molecules involved in retinal inflammatory disorders.

### ICAM-1 upregulation after retinal ischemia–reperfusion

**Figure 5:**
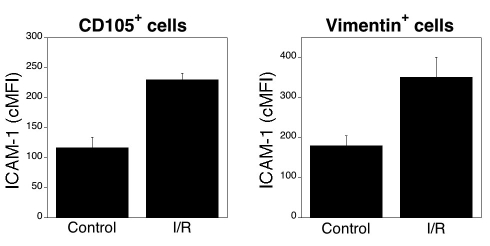
Upregulation of ICAM-1 on CD105^+^ and vimentin^+^ cells after ischemia or reperfusion. One eye from each mouse was subjected to ischemia- reperfusion (I-R). Nonischemic eyes were used as controls. Eyes were obtained 1 day after I-R. Retinal cell suspensions were stained with isotype control antibodies, anti-CD105, or anti-vimentin antibodies plus an anti-ICAM-1 antibody. Expression of ICAM-1 (cMFI) was calculated on gated CD105^+^ and vimentin^+^ cells. Data are shown as the mean±SEM from three independent experiments.

Next, we examined if flow cytometry can quantify upregulation of ICAM-1. Endothelial and Müller cells are major components of the blood-retinal barrier [25] and thus, are likely to be involved in the adhesion-dependent recruitment of leukocytes to the retina. Ischemia-Reperfusion (I-R) of the retina results in upregulation of ICAM-1 mRNA [1]. However, it is not known if ICAM-1 protein levels increase in retinal endothelial and Müller cells after I-R. We used a well established model of I/R-induced retinal injury (transient elevation of IOP) [1] to examine this possibility. Retinal cells from ischemic and nonischemic eyes were stained with isotype control antibodies or with an anti-ICAM-1 mAb plus either anti-CD105 or anti-vimentin antibodies. CD105^+^ and vimentin^+^ cells from ischemic retinas upregulated ICAM-1 compared to cells from control retinas (Figure 5; p=0.03; n=3). These results indicate that flow cytometry allows measuring upregulation of a surface molecule involved in retinal inflammatory processes.

### Upregulation of NOS2 in retinal microglia after retinal ischemia/reperfusion

**Figure 6:**
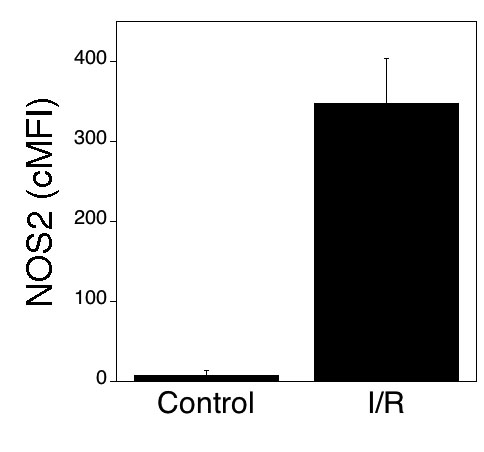
Upregulation of NOS2 in retinal CD11b^+^ cells after ischemia or reperfusion. One eye from each mouse was subjected to ischemia-reperfusion (I-R). Nonischemic eyes were used as controls. Eyes were obtained seven days after I-R. Retinal cell suspensions were stained with isotype control or an anti-CD11b mAb followed by permeabilization and staining with anti-NOS2 antibody. Expression of NOS2 (cMFI) was calculated on gated CD11b^+^ cells. Data are shown as the mean±SEM from three independent experiments.

NOS2 plays an important role in neurovascular degeneration in retinopathies including those mediated by acute ischemia and diabetes [1,7,9,26,27]. I/R-induced retinal injury results in NOS2 upregulation in microglia at 5 to 7 days post-I/R, time points where NOS2^+^ peripheral blood neutrophils (that can also express CD11b) are no longer detected in the retina [27]. We used retinas at seven days postischemia to examine whether flow cytometry can detect upregulation of NOS2. Retinal cells from ischemic and nonischemic eyes were stained with an anti-CD11b mAb followed by permeabilization and staining with an anti-NOS2 antibody. As shown in Figure 6, NOS2 was upregulated within CD11b^+^ cells from ischemic retinas (p<0.01; n=3). These results indicate that flow cytometry allows quantitation of an intracellular molecule involved in retinal inflammatory processes.

## Discussion

A complex mixture of inflammatory mediators is increasingly recognized to be important for the development of various retinopathies. We report on the use of flow cytometry to examine expression of surface and intracellular proinflammatory molecules involved in the pathogenesis of retinal disorders. This method is quantitative and utilizes antibodies against markers previously demonstrated to identify microglia/macrophages, endothelial cells, astrocytes, photoreceptors, ganglion cells, and Müller cells in the normal mouse retina [20-24]. Thus far, we have not identified an antibody that is suitable to detect amacrine neurons, cone photoreceptors, bipolar, and horizontal cells by flow cytometry. In contrast to other markers, expression of rhodopsin was bimodal. This is likely explained by the fact that the outer segments of the photoreceptors have higher concentrations of rhodopsin than the inner segment of these cells, and rhodopsin^hi^ events have morphological features consistent with outer segments. Rhodopsin^lo^ events likely represent photoreceptor cells with outer segments sheared off in which only inner segments remain.

Immunohistochemistry studies reported constitutive low-level expression of ICAM-1 in the normal retina [28,29] and upregulation of ICAM-1 in the retinal vasculature of patients with diabetes [4]. Those studies did not identify the cell types that expressed ICAM-1. Here we show that endothelial cells, Müller cells, microglia/macrophages, astrocytes, ganglion cells, and photoreceptors express ICAM-1. Of relevance, ICAM-1 has been detected in vitro in cultured glia and neurons as well as in vivo in the external limiting membrane of the retina and photoreceptor aggregates in patients with neovascular age related macular degeneration [29-31]. The latter findings suggest that ICAM-1 expression may be involved in the pathogenesis of this disease.

I-R has been shown to lead to ICAM-1 upregulation [1,3]. However, the cell types that exhibit increased ICAM-1 in response to I-R have remained unknown. Our studies revealed that both endothelial cells and Müller cells upregulate ICAM-1 after I-R. This work does not rule out ICAM-1 upregulation in other retinal cells. Upregulation of ICAM-1 is likely relevant to the pathogenesis of I-R-induced retinopathy since the level of ICAM-1 expression regulates the degree of leukocyte recruitment in inflammation.

NOS2 is involved in the pathogenesis of various retinopathies including retinal disease caused by I-R [7,8]. We report that flow cytometry can quantitate expression of intracellular NOS2 after I-R. The fact that CD11b^+^ cells become NOS2^+^ after I-R confirms the previous immunohistochemical studies that strongly suggested acquisition of NOS2 expression by microglia after I-R [27]. We were unable to detect NOS2 in Müller cells at seven days postischemia (data not shown). However, our studies do not rule out the possibility that other retinal cells acquire NOS2 expression after I-R.

The flow cytometry assay was developed to quantitate expression of molecules involved in retinal diseases and was not intended to provide an accurate assessment of the phenotypic composition of cells within the retina. Indeed, the composition of cell suspensions obtained in this study appears to differ from the phenotypic composition estimated by microscopic evaluation of the mouse retina [32]. This discrepancy could be explained by the lack of release of all retinal cells by enzymatic treatment and by cell loss after trapping cell clumps with the cell strainer used before flow cytometric analysis. In addition, it should be noted that cell fragmentation can occur during flow sorting. For example, sorting of vimentin^+^ events revealed that approximately 25% of the events appeared to represent fragments of cells as assessed by light microscopy. In addition, cellular markers should be selected carefully since certain conditions may affect the cells that express them (for example expression of CD11b or Thy-1 when neutrophils or T cells respectively infiltrate the retina). Despite these caveats, the studies performed using the model of I-R-induced retinopathy clearly indicate that flow cytometry is useful to quantitate changes in levels of expression of proinflammatory proteins in various retinal cells.

In summary, flow cytometry can readily measure expression of surface and intracellular molecules ex vivo in various retinal cells without lengthy purification protocols. While immunohistochemistry is useful for examination of topographical distribution of proteins, flow cytometry provides a methodology where protein expression can be easily quantitated and where multiple proteins can be readily examined simultaneously by using antibodies conjugated with different fluorochromes. Flow cytometry may enable future studies to improve our understanding of the regulation of retinal diseases.

## References

[1] Zheng L Gong B Hatala DA Kern TS Retinal ischemia and reperfusion causes capillary degeneration: similarities to diabetes. Invest Ophthalmol Vis Sci 2007 48 361 7 17197555 10.1167/iovs.06-0510

[2] Joussen AM Poulaki V Le ML Koizumi K Esser C Janicki H Schraermeyer U Kociok N Fauser S Kirchhof B Kern TS Adamis AP A central role for inflammation in the pathogenesis of diabetic retinopathy. FASEB J 2004 18 1450 2 15231732 10.1096/fj.03-1476fje

[3] Tsujikawa A Ogura Y Hiroshiba N Miyamoto K Kiryu J Tojo SJ Miyasaka M Honda Y Retinal ischemia-reperfusion injury attenuated by blocking of adhesion molecules of vascular endothelium. Invest Ophthalmol Vis Sci 1999 40 1183 90 10235552

[4] McLeod DS Lefer DJ Merges C Lutty GA Enhanced expression of intracellular adhesion molecule-1 and P-selectin in the diabetic human retina and choroid. Am J Pathol 1995 147 642 53 7545873 PMC1870979

[5] Miyamoto K Khosrof S Bursell SE Rohan R Murata T Clermont AC Aiello LP Ogura Y Adamis AP Prevention of leukostasis and vascular leakage in streptozotocin-induced diabetic retinopathy via intercellular adhesion molecule-1 inhibition. Proc Natl Acad Sci USA 1999 96 10836 41 10485912 10.1073/pnas.96.19.10836 PMC17969

[6] Zheng L Howell SJ Hatala DA Huang K Kern TS Salicylate-based anti-inflammatory drugs inhibit the early lesion of diabetic retinopathy. Diabetes 2007 56 337 45 17259377 10.2337/db06-0789

[7] Hangai M Yoshimura N Hiroi K Mandai M Honda Y Inducible nitric oxide synthase in retinal ischemia-reperfusion injury. Exp Eye Res 1996 63 501 9 8994353 10.1006/exer.1996.0140

[8] Kern TS Engerman RL Pharmacologic inhibition of diabetic retinopathy: aminoguanidine and aspirin. Diabetes 2001 50 1636 42 11423486 10.2337/diabetes.50.7.1636

[9] Zheng L Du Y Miller C Gubitosi-Klug RA Ball S Berkowitz BA Kern TS Critical role of inducible nitric oxide synthase in degeneration of retinal capillaries in mice with streptozotocin-induced diabetes. Diabetologia 2007 50 1987 96 17583794 10.1007/s00125-007-0734-9

[10] Grewal IS Flavell RA CD40 and CD154 in cell-mediated immunity. Annu Rev Immunol 1998 16 111 35 9597126 10.1146/annurev.immunol.16.1.111

[11] van Kooten C Banchereau J CD40–CD40 ligand. J Leukoc Biol 2000 67 2 17 10647992 10.1002/jlb.67.1.2

[12] Andrade RM Wessendarp M Subauste CS CD154 activates macrophage anti-microbial activity in the absence of IFN-γ through a TNF-α-dependent mechanism. J Immunol 2003 171 6750 6 14662879 10.4049/jimmunol.171.12.6750

[13] Andrade RM Wessendarp M Gubbels MJ Striepen B Subauste CS CD40 induces macrophage anti-*Toxoplasma gondii* activity by triggering autophagy-dependent fusion of pathogen-containing vacuoles and lysosomes. J Clin Invest 2006 116 2366 77 16955139 10.1172/JCI28796 PMC1555650

[14] Subauste CS CD154 and type-1 cytokine response: From Hyper IgM syndrome to Human Immunodeficiency Virus infection. J Infect Dis 2002 185 S83 9 11865444 10.1086/338003

[15] Mach F Schonbeck U Sukhova GK Atkinson E Libby P Reduction of atherosclerosis in mice by inhibition of CD40 signaling. Nature 1998 394 200 3 9671306 10.1038/28204

[16] Portillo JA Van Grol J Zheng L Okenka G Gentil K Garland A Carlson EC Kern TS Subauste CS CD40 mediates retinal inflammation and neuro-vascular degeneration. J Immunol 2008 181 8719 26 19050292 10.4049/jimmunol.181.12.8719

[17] Subauste CS Wessendarp M Portilllo JA Andrade RM Hinds LM Gomez FJ Smulian AG Grubbs PA Haglund LA Pathogen-specific induction of CD154 is impaired in CD4^+^ T cells from HIV-infected individuals. J Infect Dis 2004 189 61 70 14702154 10.1086/380510

[18] Tudor KS Deem TL Cook-Mills JM Novel alpha 4-integrin ligands on an endothelial cell line. Biochem Cell Biol 2000 78 99 113 10874471

[19] Andrade RM Wessendarp M Portillo JA Yang JQ Gomez FJ Durbin JE Bishop GA Subauste CS TRAF6 signaling downstream of CD40 primes macrophages to acquire anti-microbial activity in response to TNF-α. J Immunol 2005 175 6014 21 16237096 10.4049/jimmunol.175.9.6014

[20] Zeng HY Zhu XA Zhang C Yang LP Wu LM Tso MOM Identification of sequential events and factors associated with microglial activation, migration, and cytotoxicity in retinal degeneration in rd mice. Invest Ophthalmol Vis Sci 2005 46 2992 9 16043876 10.1167/iovs.05-0118

[21] Barile GR Pachydaki SI Tari SR Lee SE Donmoyer CM Ma W Rong LL Buciarelli LG Wendt T Hörig H Hudson BI Qu W Weinberg AD Yan SF Schmidt AM The RAGE axis in early diabetic retinopathy. Invest Ophthalmol Vis Sci 2005 46 2916 24 16043866 10.1167/iovs.04-1409

[22] MacKenzie D Arendt A Hargrave P McDowell JH Molday RS LOcalization of binding sites for carboxyl terminal specific anti-rhodopsin monoclonal antibodies using synthetic peptides. Biochemistry 1984 23 6544 9 6529569 10.1021/bi00321a041

[23] Maeda A Maeda T Golczak M Palczewski K Retinopathy in mice induced by disrupted all-trans-retinal clearance. J Biol Chem 2008 283 26684 93 18658157 10.1074/jbc.M804505200 PMC2546559

[24] Barnstable CJ Drager UC Thy-1 antigen: a ganglion cell specific marker in rodent retina. Neuroscience 1984 11 847 55 6146113 10.1016/0306-4522(84)90195-7

[25] Tout S Chan-Ling T Hollander H Stone J Rhe role of Muller cells in the formation of the blood-retinal barrier. Neuroscience 1993 55 291 301 8350991 10.1016/0306-4522(93)90473-s

[26] Sennlaub F Courtois Y Goureau O Inducible nitric oxide synthase mediates retinal apoptosis in ischemic proliferative retinopathy. J Neurosci 2002 22 3987 93 12019318 10.1523/JNEUROSCI.22-10-03987.2002 PMC6757641

[27] Neufeld AH Kawai S Das S Vora S Gachie E Connor JR Manning PT Loss of retinal ganglion cells following retinal ischemia: the role of inducible nitric oxide synthase. Exp Eye Res 2002 75 521 8 12457864 10.1006/exer.2002.2042

[28] Hughes JM Brink A Witmer AN Hanraads-de Riemer M Klaassen I Schlingemann RO Vascular leucocyte adhesion molecules unaltered in the human retina in diabetes. Br J Ophthalmol 2004 88 566 72 15031178 10.1136/bjo.2003.021204 PMC1772105

[29] Mullins RF Skeie JM Malone EA Kuehn MH Macular and peripheral distribution of ICAM-1 in the human choriocapillaris and retina. Mol Vis 2006 12 224 35 16604055

[30] Héry C Sébire G Peudenier S Tardieu M Adhesion to human neurons and astrocytes of monocytes: the role of interaction of CR3 and ICAM-1 and modulation by cytokines. J Neuroimmunol 1995 57 101 9 7706427 10.1016/0165-5728(94)00168-n

[31] Shelton MD Kern TS Mieyal JJ Glutaredoxin regulates nuclear factor kappa-B and intracellular adhesion molecule in Muller cells: model of diabetic retinopathy. J Biol Chem 2007 282 12467 74 17324929 10.1074/jbc.M610863200

[32] Jeon CJ Strettoi E Masland RH The major cell populations of the mouse retina. J Neurosci 1998 18 8936 46 9786999 10.1523/JNEUROSCI.18-21-08936.1998 PMC6793518

